# How cognitive loads modulate the postural control of older women with low back pain?

**DOI:** 10.1186/s12877-021-02025-z

**Published:** 2021-01-28

**Authors:** Le Ge, Qiuhua Yu, Chuhuai Wang, Huanjie Huang, Xin Li, Shanshan Zhang, Siyun Zhang

**Affiliations:** Department of Rehabilitation Medicine, The First Affiliated Hospital, Sun Yat-sen University, Zhongshan Road 2, Guangzhou, 510080 Guangdong Province China

**Keywords:** Low back pain, Older women, Postural control, Cognitive load, Balance

## Abstract

**Background:**

The capacity of postural control is a key factor related to falling in older people, particularly in older women with low back pain (LBP). Cognitive involvement in postural control increases with age. However, most scholars have not considered different difficulty levels of cognitive loads when exploring the effects of cognition on postural control in older patients with LBP. The present study is to investigate how different levels of cognitive loads modulate postural control in older women with LBP.

**Methods:**

This was a cross-sectional study. Twenty older women with LBP were recruited into the LBP group, and 20 healthy older women without the history of LBP were recruited into the healthy control group. Balance parameters were computed to quantify postural control. All participants underwent the balance test, which required the participant to maintain stability during standing on a force platform with or without a concurrent cognitive task. The balance test included three levels of difficulties of posture tasks (eyes-open vs. eyes-closed vs. one-leg stance) and three cognitive tasks (without cognitive task vs. auditory arithmetic task vs. serial-7 s arithmetic task).

**Results:**

A repeated-measure analysis of variance (3 postural tasks × 3 congnitive tasks× 2 groups) testing the effects of the different congnitive task levels on the performance in different postural conditions. Older women with LBP had worse postural control (as reflected by larger center of pressure (COP) parameters) than control group regardless of postural or cognitive difficulties. Compared with the single task, the COP parameters of participants with LBP were larger during dual tasks, even though the difficulty level of the cognitive task was low. Larger COP parameters were shown only if the difficulty level of the cognitive task was high in control group. Correlations between sway area/sway length and the number of falls were significant in dual tasks.

**Conclusion:**

Our findings shed light on how cognitive loads modulate postural control for older women with LBP. Compared with control group, cognitive loads showed more disturbing effects on postural control in older women with LBP, which was associated with falling.

## Background

It has been reported that around one-third of older people (age > 60 years) have a high risk of falling (falling at least once a year) [1]. Poor postural control (PC) is a key factor for falling in older people [2]. Studies have shown that cognition can modulate PC [3] and that the modulation effect increases with aging [4]. In daily life, it is very common for postural tasks to be accompanied with cognitive tasks (e.g., making a telephone call while walking). In such situations, attentional resources must be divided to undertaken both tasks appropriately [5].

Studies have shown that decreased/divided attention is a high risk factor for falling in daily life for older people [6, 7]. This finding was supported by a large number of studies, which explored the effect of dual-tasking on postural control in older people [8–10]. The findings of these studies showed that older people had poor performance in maintaining motor patterns or PC in dual tasks, in which postural and cognitive tasks must be completed simultaneously. For instance, Brauer and colleagues [11] reported postural stability to be impaired among older participants with a history of falling as compared with that in healthy counterparts while undertaking a dual task (verbal reaction to an auditory-tone task). If one of the dual tasks needs a high level of arousal or increased attentional demand, not sufficient cognitive resources may be able to be allocated to carry out daily activities (e.g., undertaking functional activities and maintaining postural balance simultaneously), thereby leading to higher risk of falling. The potential reason was that the concurrent postural and cognitive tasks would compete with each other for the cognitive resources, leading to a further degradation in the performance of both tasks. However, Huxhold and coworkers [12] showed that older people have enhanced postural stability in a dual task, of which the cognitive task is easy. Their findings mentioned above are in accordance with the “U-shaped” relationship between PC and cognitive demands [13]. All the findings in the previous studies suggested that high cognitive demands seem to have inhibited effect upon PC in dual-task conditions, whereas the low cognitive demands have facilitative effect upon PC in dual-task conditions [5].

A prospective study showed that older people in the community with low back pain (LBP) had a significantly higher risk of falling than older people without pain [14]. LBP, which is more common in females than males [15], is known to be an important risk factor for repeated falls in older women [16]. The older women with LBP, who suffer from both low back pain and aging-related cognitive decline, seem to be more susceptible to falling. Thus, investigating the modulation of cognitive loads to postural control was very important for the older women with low back pain, which could help understanding of the mechanism underlying falling for older women with LBP. Studies have demonstrated that older people with LBP may have impaired PC compared with healthy older adults [15–17]. However, adoption of only a single postural task is not sufficient to explain PC ability in daily lives, which requires completion of multiple tasks simultaneously. Some studies have shown that higher cognitive loads could reduce the PC of people with LBP in a dual-task paradigm. For instance, Etemadi et al. [18] found that a LBP group had worse PC and cognitive performance under a dual-task condition than that of a control group. Nevertheless, Salavati et al. [19] found that the PC of participants with LBP did not differentiate with that of a control group in the dual-task condition, but the cognitive performance of LBP group was impaired in the postural task with higher difficulty. There may be two reasons for these inconsistent findings: (i) those studies did not take different difficulty levels of postural tasks and cognitive tasks into consideration;(ii) the sample population in those dual-task studies were young and middle-aged people with LBP. For older people, contradictory results in bipedal standing have been shown in participants with LBP compared with that in healthy controls [20, 21]. However, little is known about the modulation of cognitive loads to PC in older women with LBP. In the present study, we employed different levels of difficulties for postural tasks and cognitive tasks to explore how cognitive loads modulate the PC in older women with LBP assessed under single-task and dual-task conditions. We hypothesized that poorer PC performance would be observed in the dual task than single task in LBP group compared to the healthy control group. The more difficult the postural / cognitive task is, the poorer PC performance is. It was also anticipated that cognitive performance was poor when the postural task was difficult.

## Methods

### Participants

Twenty patients with LBP (age = 64.90 ± 3.33 years, mean ± SD) and 20 healthy participants as control group (age = 63.20 ± 2.33 years, mean ± SD) were recruited from local community and different older activity centers by posting the advertisement. The study protocol was approved by the ethics committee of the First Affiliated Hospital of Sun Yat-sen University (grant number#2019469) in Guangzhou, China, in accordance with the Declaration of Helsinki and informed consent was obtained from individuals prior to participation. The inclusion criteria for the LBP group were: (i) female, aged of ≥60 years; (ii) nonspecific LBP for ≥3 months in the previous year; (iii) the worst pain during the previous 3 months rated 3 to 10 (out of 10) on a visual analog scale (VAS); (iv) a Mini-Mental State Examination (MMSE) score > 24 (out of 30) and Montreal Cognitive Assessment (MoCA) score ≥ 26 (out of 30). The inclusion criteria for the control group were: (i) female, age ≥ 60 years; (ii) a Mini-Mental State Examination (MMSE) score > 24 (out of 30) and Montreal Cognitive Assessment (MoCA) score ≥ 26 (out of 30); (iii) had no history of low back pain for a minimum of 1 year. Participants in both LBP and control groups were excluded if any of the following criteria were met: (i) a history of spinal or low-extremity surgery, traumatic event, endocrine/neuromuscular disease, spinal tumor, rheumatologic disease of the spine, arthritis or orthopedic disease, orthostatic hypotension, vision, vestibular-system disease or any other physical injury that might affect balance; (ii) use of psychoactive or antihypertensive drugs (antidepressants, antipsychotics, sedatives/ hypnotics, antiepileptics, antiparkinsonian drugs); (iii) severe posture abnormalities.

### Instruments and experimental design

**Fig. 1:**
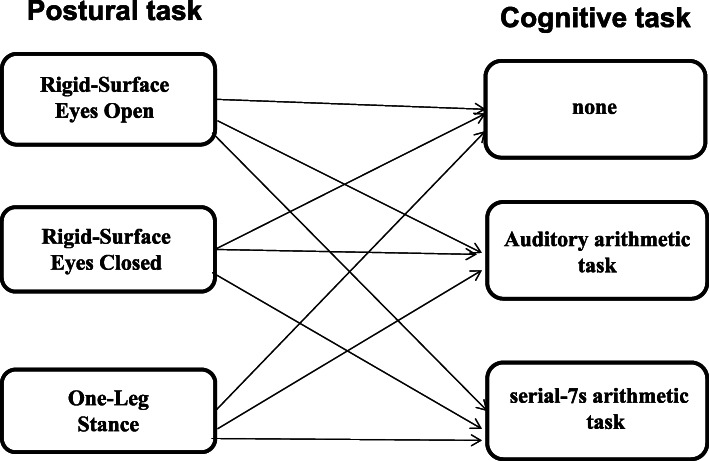
Combinations of postural tasks and cognitive tasks

PC was measured by the center of pressure (COP) in two conditions, which were single task (only a postural task) and dual task (carrying out the postural task with a concurrent cognitive task) (Fig. 1). All the conditions in single and dual tasks were assigned randomly to participants and the order was determined by the random function in the Microsoft Office Excel 2007. Each condition was repeated thrice, with each lasting for 30s.

### Postural task

**Fig. 2:**
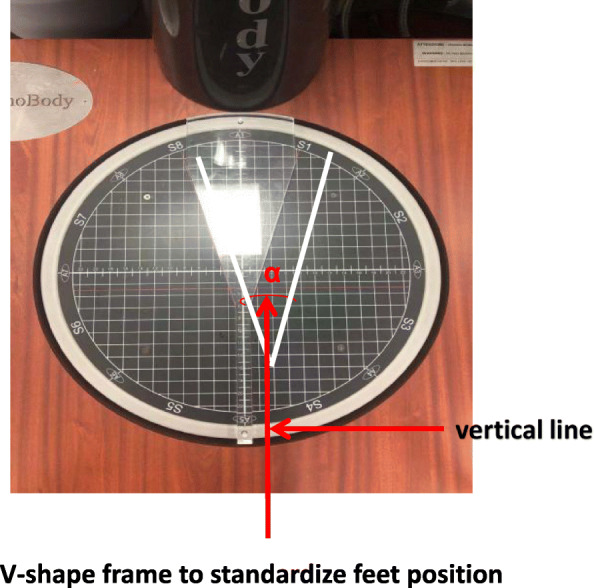
V-shape frame to standardize feet position. Imagine in this figure belongs to our own work

The postural task required a participant to stand barefoot on a force platform of TecnoBody system (PK254P; TecnoBody, Italy) with his/her arms hanging by the side. This task could be divided into three conditions of different difficulty while standing on the force platform: (i) with eyes open; (ii) with eyes closed; (iii) taking a one-leg stance. During the eye-open condition, the participant was asked to look straight ahead to the white wall 80 cm in front of the participant’s eyes. In both eyes-open and eyes-closed conditions, the position of the feet on the platform was standardized using a V-shaped frame (Fig. 2). The participants had to place the medial borders of the feet against the frame; the malleolus were aligned to vertical line. The distance between one malleolus and the other was 3 cm. The medial borders of the feet were extra-rotated 12° with respect to the anteroposterior axis. In one-leg stance condition, participant was required to practice the one-leg stance before testing and choose which leg he/she preferred to stand on [22], since LBP people prefer to choose non-painful side. COP displacements during the tasks were recorded by TecnoBody system (PK254P; TecnoBody, Italy). All the COP signals were sampled at a rate of 100 Hz and filtered at 8 Hz (by a 30th order low-pass FIR filter with zero-phase) and down-sampled at 20 Hz [23]. The COP parameters involving sway length (mm), sway area (mm^2^), anteroposterior (AP) velocity (mm/s) and mediolateral (ML) velocity (mm/s), which could be computed by the TecnoBody system. Each condition was repeated thrice, with each lasting for 30s. Three conditions were assigned randomly to participants. Before testing, participants were required to stand on the force platform to become familiar with the test environment, and to select the most suitable standing leg for testing.

### Cognitive task

The cognitive task consisted of two subtasks with high difficulty and low difficulty. The subtask with low difficulty was an auditory arithmetic task (Task 1). In the auditory arithmetic task, the participant was required to complete the calculation by an auditory stimulus. The auditory stimulus (including 93–7 =?,79–7 =?,100–7 =?,86–7 =?, and 72–7 =?) were displayed in a randomized order. The participant was asked to give an answer as soon as possible. The other subtask with high difficulty was a “serial-7s arithmetic task” (Task 2), making reference to the task in the study by Swanenburg and colleagues [24]. In the serial-7 s arithmetic task, the participant was required to start with 100, then subtract 7 several times within 30s. The participant was asked to give an answer as fast as possible when subtracting 7 at each time. So the participant need to remember the answer for the last equation and continue to subtract 7. This was different from the auditory arithmetic task, which did not require the participant to remember the answer for the last equation. The percentage accuracy in all cognitive tasks was used in subsequent data analyses.

Each participant also undertook two calculation tasks sitting in a chair with eyes-open or with eyes-closed at the beginning of testing. The purpose of the calculation tasks in the sitting position was to ensure that each participant could complete the cognitive tasks. The cognitive performance in the sitting position was used in data analyses as the baseline of cognitive performance [25].

### Procedure

**Fig. 3:**
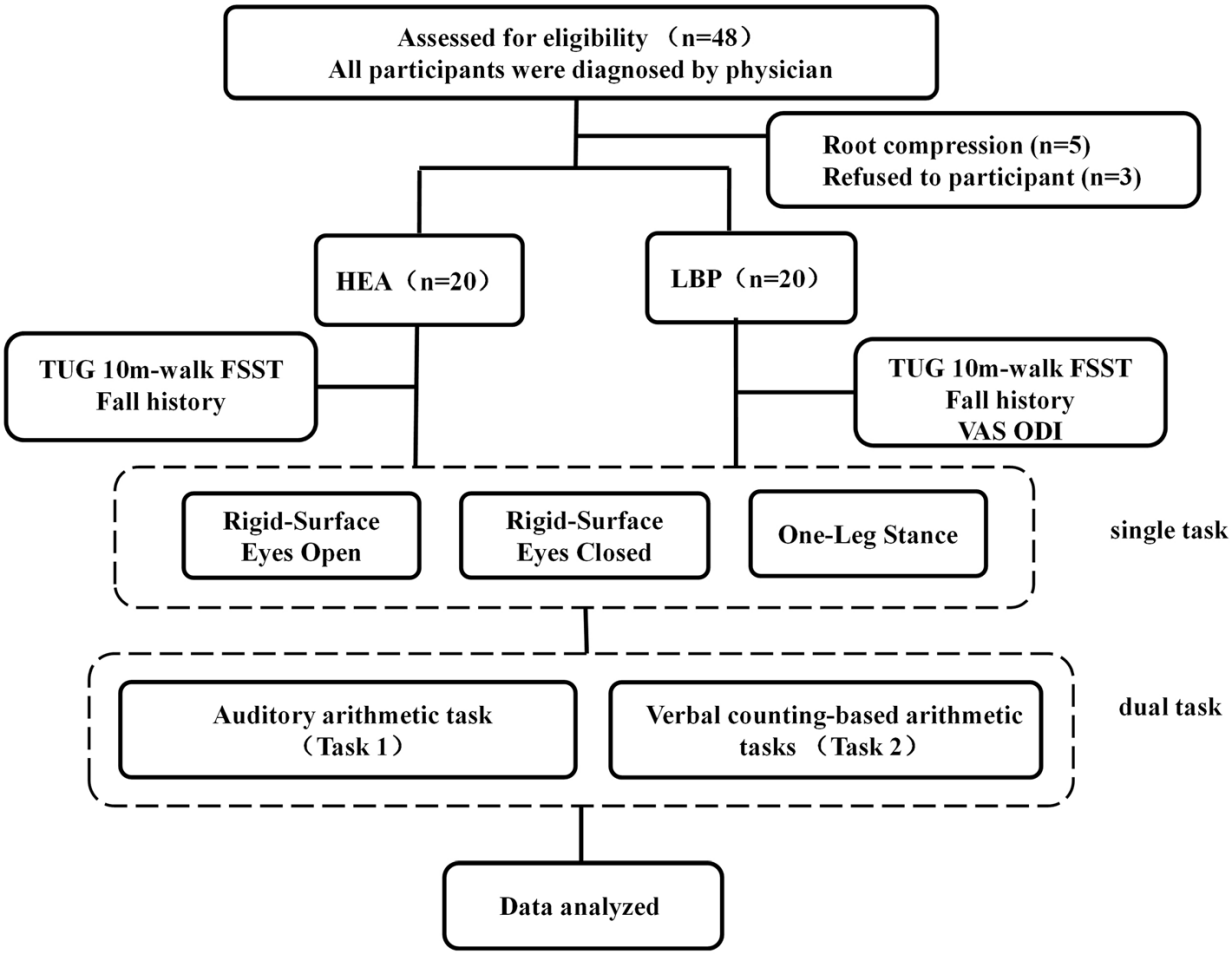
Flowchart showing participant screening and the experimental protocol

The whole experiment took ~ 1 h. At the beginning of the experiment, sociodemographic data, education level, abdominal circumference, and the number of falls in the previous year were recorded in an individual information sheet. Data on weight, height, body mass index (BMI) and abdominal circumference were also obtained during the experiment. The history of falling was recorded in the individual information sheet. We defined a “fall” as unintentionally coming to rest on the ground, floor, or other level with or without an injury. Participants with LBP also completed four questionnaires: 10-cm VAS; Oswestry Disability Index (ODI); MMSE; MoCA. The postural control assessments were conducted in a brightly lit, safe and quiet physiotherapy room. The mean and SD of all COP parameters (AP velocity, ML velocity, sway area, sway length) were used in data analyses. If participants could not complete a single task, they did not receive a dual-task assessment (Fig. 3).

### Statistical analysis

Descriptive statistics were used to describe demographics. The independent *t*-test was employed to determine the differences in age, height, weight, MMSS, MoCA, BMI and Abdominal circumference between LBP and control groups. Chi-square test was employed to determine the between-group difference in the falls in the past 12 months. In the present study significant between-group differences, were showed in both BMI and abdominal circumference (Table 1). Because these two parameters were associated with cognition and balance [26, 27], so BMI and abdominal circumference were used as covariates in the statistical analysis. By adjusting the covariates of BMI and abdominal circumference, the data of postural and cognitive performance in the single or dual tasks were assessed using a mixed model analysis of covariance. For the postural performance, the within-participant factors were postural difficulties (eyes-open, eyes-closed, or one-leg stance) and cognitive difficulties (none, auditory arithmetic task, or serial-7 s arithmetic task), and the between-participant factor was group (LBP or control group). Dependent variables were the mean values of three trials of all COP parameters in each condition and cognitive-task accuracy. For the cognitive performance, the within-participant factors were postural difficulties (eyes-open, eyes-closed, or one-leg stance) and cognitive difficulties (auditory arithmetic task, or serial-7 s arithmetic task), and the between-participant factor was group (LBP or control group). Dependent variables were the accuracy rates in cognitive tasks of different postural tasks. Post hoc pairwise comparisons with the Bonferroni adjustment were applied for significant main or interaction effects. The Greenhouse–Geisser correction was used if Mauchly’s test of sphericity was violated. Analysis of covariance (ANCOVA) with the covariates of BMI and abdominal circumference was conducted to test cognitive performance in the single or dual tasks. Studies have shown that the sway length and sway area are valid fall-risk predictors and a holistic analysis of postural stability [28, 29]. The sway area and sway length were selected to explore the associations between the COP parameters and the number of falls. *P* < 0.05 was considered significant in all statistical tests. Data were analyzed using SPSS v23.0 (IBM, Armonk, NY, USA).

**Table 1:** Demographic characteristics of the two groups

Characteristic	LBP (*n* = 20)	control group (*n* = 20)	*t*	*P*
Age (years)	64.90 (3.33)	63.20 (2.33)	0.87	0.38
Height (m)	1.57 (0.04)	1.58 (0.03)	−0.28	0.77
Weight (kg)	58.25 (5.18)	55.80 (3.54)	1.74	0.89
MMSE	29.05 (0.94)	29.00 (0.97)	0.16	0.87
MoCA	26.65 (0.87)	27.20 (1.19)	−1.65	0.10
Body mass index (kg/m^2^)	23.43 (1.97)	22.34 (1.17)	2.15	0.03
Abdominal circumference (cm)	87.40 (8.78)	81.20 (3.17)	2.96	0.05
Pain duration (years)	13.40 (9.90)	Not applicable		
VAS rating	8.00 (2)	Not applicable		
ODI(%)	30.00 (17.5)	Not applicable		
Falls in the past 12 months	0(8)	0(14)	–	0.04
	1(8)	1(5)	–	
	2(4)	2(1)	–	

Noted: VAS and ODI, are shown as median (interquartile range); the falls in the past12 months are expressed as number of falls (number of person);other outcome variables are shown as mean (standard deviations)

*LBP* low back pain, *MMSE* Mini-Mental State Examination, *MoCA* Montreal Cognitive Assessment, *VAS* visual analog scale, *ODI* Oswestry disability index

## Results

### Participants

Twenty patients with LBP (LBP group) and twenty healthy individuals (control group) were recruited in the present study. All study participants were female and right-handed. There were no significant differences in age, weight, or height between two groups (*P* ≥ 0.05 for all) (Table 1). However, the LBP group had significantly higher BMI and abdominal circumference, and more falls in the previous 12 months (Table 1).

### Postural performance in the single or dual tasks

Five participants in the LBP group could not complete the one-leg stance in a single task or dual task, so the data of 15 participants with LBP were used in the mixed model repeated-measure ANCOVA. Table 2 shows the mean (SD) of COP parameters in different combinations of postural difficulty and cognitive difficulty. Table 3 presents a summary of ANCOVA results for all data of postural performance in the single or dual tasks.

**Table 2:** Performance in different combinations of postural difficulty and cognitive difficulty for the two groups

Conditions		AP velocity Mean (SD)		ML velocity Mean (SD)		Sway area Mean (SD)		Sway length Mean (SD)	
		LBP	Control	LBP	Control	LBP	Control	LBP	Control
Single task	Eyes-open	10.80 (2.48)	9.85 (2.81)	7.67 (2.10)	6.55 (2.37)	228.07 (60.157)	204.85 (65.07)	309.20 (78.18)	302.55 (65.73)
	Eyes-closed	15.13 (3.29)	14.50 (3.67)	10.53 (2.23)	10.65 (4.06)	403.73 (85.68)	382.50 (123.51)	498.73 (74.48)	527.85 (157.63)
	One-leg stance	29.80 (8.81)	23.50 (5.54)	27.53 (6.44)	17.95 (4.39)	973.33 (231.23)	720.75 (261.76)	1216.00 (203.46)	881..35 (223.15)
Dual task	Eyes-open + task 1	12.67 (2.09)	8.25 (1.37)	8.73 (1.90)	5.75 (1.02)	388.27 (75.48)	150.80 (44.30)	427.00 (86.19)	253.85 (52.77)
	Eyes-closed + task 1	16.67 (1.67)	11.60 (1.63)	13.07 (2.05)	9.05 (1.63)	527.80 (96.17)	316.55 (68.39)	558.73 (51.347)	412.65 (94.09)
	One-leg stance + task 1	34.40 (4.98)	27.10 (3.72)	32.80 (5.03)	24.35 (3.99)	1353.80 (330.22)	892.80 (196.00)	1190.33 (298.58)	969.75 (152.37)
	Eyes-open + task 2	19.53 (3.24)	16.05 (3.15)	14.20 (2.24)	12.30 (2.71)	563.93 (113.92)	291.95 (79.60)	600.87 (127.38)	413.55 (123.30)
	Eyes-closed + task 2	21.20 (3.38)	18.10 (1.83)	15.87 (1.72)	13.85 (1.78)	698.27 (73.87)	383.05 (72.04)	703.93 68.56)	501.20 (55.02)
	One-leg stance + task 2	38.27 (6.71)	31.90 (3.82)	35.20 (4.66)	28.00 (2.73)	1647.47 (299.05)	1227.65 (243.06)	1506.00 (240.32)	1213.30 (198.42)

Noted: SD denotes standard deviation

*AP* anteroposterior, *ML* mediolateral, task 1: auditory arithmetic task, task 2: serial-7 s arithmetic task

**Table 3:** Summary of F and *P* values for four COP parameters

Independent variable	AP velocity		ML velocity		Sway area		Sway length	
	*F*	*P*	*F*	*P*	*F*	*P*	*F*	*P*
Main effect								
Group	49.260	< 0.001	115.997	< 0.001	82.414	< 0.001	48.600	< 0.001
Postural difficulty	8.254	0.004	3.920	< 0.001	1.662	0.207	3.128	0.069
Cognitive difficulty	0.192	0.778	2.554	0.085	0.518	0.582	0.415	0.653
Interaction effect								
Group × postural difficulty	6.862	0.008	17.359	< 0.001	9.142	0.003	7.198	0.005
Group × cognitive difficulty	2.994	0.070	3.448	0.038	15.065	< 0.001	5.346	0.007
Postural × cognitive difficulty	0.527	0.654	1.001	0.387	0.536	0.610	1.682	0.183
Group × postural × cognitive difficulty	0.794	0.494	0.566	0.609	0.596	0.575	3.044	0.040

Noted: *AP* anteroposterior, *ML* mediolateral

With regard to balance performance, the covariate of BMI AP:[*F*(1,31) = 1.122, *P* = 0.298,ƞ2p = 0.035], ML:[*F*(1,31) = 0.076,*P* = 0.784,ƞ2p = 0.002], sway area:[*F*(1,31) = 0.115, *P* = 0.737,ƞ2p = 0.004], sway length:[*F*(1,31) = 0.454, *P* = 0.505,ƞ2p = 0.014] and abdominal circumference AP: [*F*(1,31) = 1.039, *P* = 0.316,ƞ2p = 0.032], ML: [*F*(1,31) = 0.516 *P* = 0.478,ƞ2p = 0.015], sway area: [*F*(1,31) = 0.003, *P* = 0.956,ƞ2p = 0.000], sway length:[*F*(1,31) = 0.010,*P* = 0.920,ƞ2p = 0.000] were not significant in all the COP parameters. The COP parameters were significant between two groups AP:[*F*(1,31) = 49.260, *P* < 0.001,ƞ2p = 0.614], ML:[*F*(1,31) = 115.997, *P* < 0.001,ƞ2p = 0.789], sway area: [*F*(1,31) = 82.414, *P* < 0.001,ƞ2p = 0.727], sway length:[*F*(1,31) = 48.600, *P* < 0.001,ƞ2p = 0.611]. The main effects of postural difficulty were significant in AP:[*F*(1.297,40.197) = 8.254, *P* < 0.005,ƞ2p = 0.210],ML:[*F*(1.202,37.250) = 3.920, *P* < 0.001,ƞ2p = 0.112], and sway length:[*F*(1.425,44.175) = 3.128, *P* = 0.069, ƞ2p = 0.092], but not significant in sway area:[*F*(1.160,35.972) = 1.662, *P* = 0.207, ƞ2p = 0.051]. The main effects of cognitive difficulty were not significant in all COP parameters AP: [*F*(1.607,49.823) = 0.192, *P* = 0.778, ƞ2p = 0.006], ML:[*F*(2,62) = 2.554, *P* = 0.086,ƞ2p = 0.076],sway area:[*F*(2,62) = 0.518, *P* = 0.582,ƞ2p = 0.016 and sway length:[*F*(2,62) = 0.415, *P* = 0.653,ƞ2p = 0.013].

**Fig. 4:**
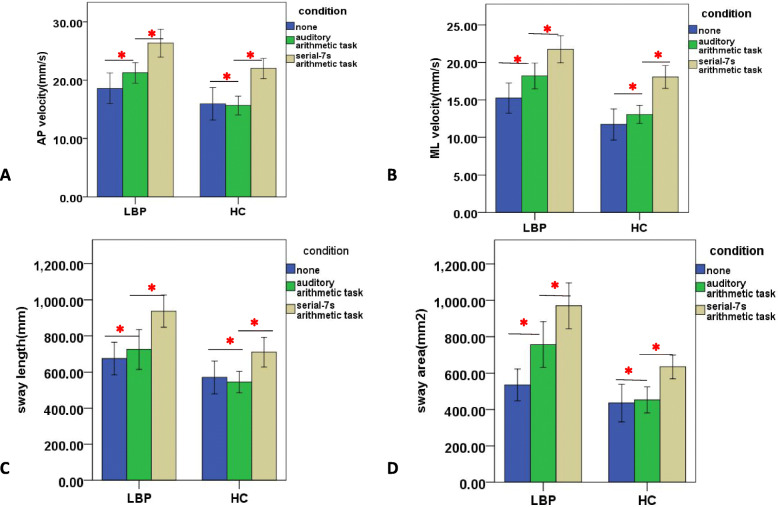
The results of post hoc analysis for the cognitive difficulty × group effects. Noted:error bar denotes ±SD;AP: anteroposterior; ML: mediolateral; LBP: low back pain; HC: control group; The significant results (*P* < 0.05) of multiple comparisons of means are shown with an red asterisk

The group × postural difficulty × cognitive difficulty effect was only significant in sway length:[*F*(2.615,81.067) = 3.044, *P* = 0.040,ƞ2p = 0.089]. Post hoc analysis showed that the LBP group had larger sway length than the control group in the dual task (*P* < 0.05) but not in the single task, when standing on a force platform with eyes open or closed. But the LBP group showed larger sway length than the control group in both the single and dual tasks (*P* < 0.05) in one-leg stance. The group × postural difficulty effects were significant in all COP parameters AP: [*F*(1.297,40.197) = 6.862, *P* = 0.008,ƞ2p = 0.181],ML:[*F*(1.202,37.250) = 17.359,*P* < 0.001,ƞ2p = 0.435], sway area:[*F*(1.160,35.972) = 9.142, *P* = 0.003,ƞ2p = 0.228] and sway length: [*F*(1.425,44.175) = 7.198, *P* = 0.005, ƞ2p = 0.188]. Post hoc analysis showed that the LBP group had larger COP parameters than the control group in three tasks with different postural difficulties (*P* < 0.05). The cognitive difficulty × group effects were significant in ML:[*F*(2,62) = 3.448, *P* = 0.038, ƞ2p = 0.100], sway area: [*F*(2,62) = 15.065, *P* < 0.001,ƞ2p = 0.327], sway length:[*F*(2,62) = 5.346, *P* = 0.007, ƞ2p = 0.147], and marginally significant in AP: [*F*(1.607,49.823) = 2.994, *P* = 0.070, ƞ2p = 0.089]. Post hoc analysis for the cognitive difficulty × group effects showed that the LBP group had larger COP parameters than the control group in three tasks with different cognition difficulties (*P* < 0.05). LBP participants showed larger COP parameters in the dual tasks with high and low cognitive difficulties than those in single task (*P* < 0.05), whereas the control participants only displayed larger COP parameters in the dual task with higher cognitive difficulty than those in single task (*P* < 0.05)(Fig. 4). Both LBP and control participants showed larger COP parameters in the dual task with higher cognitive difficulty that the dual task with lower cognitive difficulty (*P* < 0.001). No significant postural difficulty × cognitive difficulty effects were found in COP parameters. These results suggested that compared to the healthy older people, the older people with LBP had poor postural performance reflected by larger COP parameters regardless of any postural or cognitive difficulties. Compared with the single task, the LBP participants’ postural control were decreased in the dual task, even though the difficulty level of the cognitive task was low. The control participants’ postural balance, however, were poor when the difficulty level of the cognitive task was high. The postural balance of control groups, however, was decreased when the difficulty level of the cognitive task was high.

### Cognitive performance in the postural tasks

The cognitive performance results in the postural tasks are shown in Table 4.The covariates of BMI (*F* = 7.258, *P* = 0.011), and abdominal circumference (*F* = 11.123, *P* = 0.002) were significant in the condition of auditory arithmetic task with a one-leg stance. BMI and abdominal circumference, however, were not significant in other conditions (*P* ≥ 0.05). After adjustment of the covariates of BMI and abdominal circumference, the group main effect (*F* = 6.011, *P* = 0.019) and the interaction effect of group × postural difficulty (*F* = 6.859, *P* = 0.003) were significant, whereas other main effects and interaction effects were not significant (Table 4). Post hoc analyses for the interaction effect of group × postural difficulty showed that the LBP group had lower percentage accuracy than that of the control group only in the one-leg stance condition (*P* < 0.05) (Table 5). However, there was no significant between-group difference in two-leg stance with eyes-open or eyes-closed (*P* ≥ 0.05).

**Table 4:** The summary of statistical results for accuracy rates of cognitive tasks with different postural tasks

Independent variable	*F*	*P*	ƞ^2^p
Main effect			
Group	6.011	0.019	0.143
postural difficulty	0.889	0.415	0.024
cognitive difficulty	3.704	0.062	0.093
Interaction effect			
group × postural difficulty	6.859	0.003	0.160
group × cognitive difficulty	0.373	0.545	0.010
postural × cognitive difficulty	2.067	0.141	0.054
group × postural × cognitive difficulty	1.231	0.295	0.033

**Table 5:** The accuracy rates of cognitive tasks with different postural tasks

	Cognitive performance(%)			
	LBP (*n* = 20) Mean (SD)	control group (*n* = 20) Mean (SD)	*t*	*P*
Eyes-open + task 1	94 (9)	96 (6)	0.011	0.916
Eyes-closed + task 1	98 (4)	98 (3)	0.810	0.374
One-leg stance + task 1	83 (7)	93 (7)	10.978	0.002
Eyes-open + task 2	91 (14)	91 (12)	0.383	0.540
Eyes-closed + task 2	98 (5)	96 (9)	0.941	0.339
One-leg stance + task 2	76 (18)	88 (15)	6.316	0.017

Noted: SD denotes standard deviation

### Associations between COP parameters and the number of falls

The associations between the COP parameters in all conditions and the number of falls are shown in Table 6. Significant associations between sway area (*R* = 0.386)/sway length (*R* = 0.482) and the number of falls in a single task were shown only in the eye-closed condition (*P* < 0.05). The correlations between sway area and the number of falls in dual task 1 (postural task and auditory arithmetic task) were significant in eyes-open (*R* = 0.314) and eyes-closed (*R* = 0.323) conditions (*P* < 0.05). The correlations between sway length (*R* = 0.445,*R* = 0.331,*R* = 0.347) and the number of falls in dual task 2 (postural task and serial-7 s arithmetic task) were significant in the eyes-open, eyes-closed, and one-leg stance conditions(*P* < 0.05). The other associations between the COP parameters and the number of falls were not significant(*P* ≥ 0.05).

**Table 6:** Relationship between COP parameters in all conditions and the number of falls

		SO_sway area	SO_sway length	SC_sway area	SC_sway length	Sleg_sway area	Sleg_sway length	DO1_sway area	DO1_sway length	DC1_sway area	DC1_sway length	Dleg1_sway area	Dleg1_sway length	DO2_sway area	DO2_sway length	DC2_sway area	DC2_sway length	Dleg2_sway area	Dleg2_sway length
the number of falls	rho	0.281	0.217	0.386*	0.482**	0.089	0.277	0.314*	0.267	0.323*	0.212	0.031	0.048	0.269	0.445**	0.221	0.331*	0.288	0.347*
	*P*	0.079	0.179	0.014	0.002	0.605	0.102	0.048	0.096	0.042	0.189	0.858	0.782	0.093	0.004	0.171	0.037	0.088	0.038

*SO* single task with eyes-open, *SC* single task with eyes-closed, *Sleg* single task with one-leg stance, *DO1* dual task (postural task and auditory arithmetic task) with eyes-open, *DC1* dual task (postural task and auditory arithmetic task) with eyes-closed, *Dleg1* dual task (postural task and auditory arithmetic task) with one-leg stance, *DO2* dual task (postural task and Serial-7 s arithmetic task) with eyes-open, *DC2* dual task (postural task and Serial-7 s arithmetic task) with eyes-closed, *Dleg2* dual task (postural task and Serial-7 s arithmetic task) with one-leg stance

## Discussion

In the present study, the modulation effect of cognitive loads on the PC of older women with LBP was examined. Posture performance was meet our expectations: the more difficult the postural / cognitive task is, the poorer PC performance is. That is to say, participants performed worst in one-leg stance and performed best in the eyes-open condition of three posture conditions. Load manipulations were successful in all experiments: participants’ performed worse in the high-load task (task 2) when compared to the low-load task (task 1). When cognitive task combined with postural task, the results was complicated. These results were consistent with our hypothesis. Compared with the single task, the PC of participants with LBP became worse in the dual task, even though the difficulty level of the cognitive task was low. The PC of control group, however, was decreased only if the difficulty level of the cognitive task was high. The performance in the LBP group were supported by Sherafat et al. [30] They found significant differences between single-task and dual-task (auditory Stroop test as the concurrent cognitive) conditions in adult patients with LBP. Sherafat et al’s study, however, did not employ different difficult levels of cognitive tasks. The potential reason for the dissociated performance of older LBP patients from the control group’s performance was that the presence of pain may require attention and may compete for limited attentional resources [31]. Previous studies have shown pain was associated with poorer cognitive functioning in the domains of memory, mental flexibility, emotional decision making, and attention [32, 33]. For instance, Weiner et al. [34] found cognitive performance in older adults with LBP to be lower than that in healthy older adults. In this study LBP group’s attention capacity was reduced due to long-term pain. Thus, it was not enough attentional resources to complete PC task in dual tasks for the participants with LBP, sequentially leading to poor balance performance or motor behavior. Nevertheless, the deficits in balance of healthy older people could be compensated by cognitive system in the dual task when the cognitive task was not difficult.

A decreased PC performance of healthy older people was only found at a high difficulty level of the cognitive task, which was consistent with those reported by Huxhold and coworkers [12]. They reported the postural stability of healthy older people to be enhanced in the dual-task with a digit choice reaction time task as the less difficult cognitive task than that in a single task, whereas those postural stability were reduced in the dual task with digit and spatial 2-back memory tasks as the more difficult cognitive task than single task. Marchese and colleagues [35], for instance, reported the inhibitory effects of verbal serial-7 s arithmetic tasks (counting backwards aloud in multiples of three) on postural stability. Conversely, Mak and collaborators [36] reported a facilitation effect of nonverbal tasks (auditory switch task) on the postural stability of healthy older participants. Our findings in the control group were supported by the U-shaped model proposed by Lacour and coworkers^5^ to explain the relationship between PC and cognitive demand. In the U-shaped model, posture stability could be modulated by the consumption of attentional resources of the second task. If the second task requires a lower level of attentional resources, postural stability will increase for healthy participants. This phenomenon may be due to a shift in the focus of attention away from PC, thereby increasing the automatic processing of posture [37, 38]. However, if the consumption of attentional resources of the second task increases, postural stability would be reduced due to the limited capacity of the brain. In our study, the PC deficits of healthy older people were not observed in the dual task with a less difficult cognitive task due to the compensation from higher cognitive systems to a certain extent. However, the low-load attention task seemed to disturb, rather than facilitate, PC for older patients with LBP due to their poor PC performance.

We also showed that, compared with healthy older people, older people with LBP had poor PC as reflected by larger COP parameters regardless of postural or cognitive difficulties. These findings are consistent with our hypothesis and supported by the work of Mazaheri and colleagues [39]. They reported a LBP group to have worse PC (as reflected by larger postural sway) than that of control group in a dual task (two-leg stance and counting digits). Even though the sample population in their study was adult patients with LBP, the capacity of sensory, motor, and cognitive processing decreases with aging [40, 41]. For instance, Lee and coworkers [21] investigated postural responses to sudden release of a pulling force in older adults with and without LBP, the LBP group had worse PC than that of control group. Thus, compared with PC in control group, the poor PC of older participants with LBP was most likely due to decreased motor and cognitive functions [42].

The cognitive performance of the LBP group in the postural tasks was poor compared with that in the control group only in the one-leg stance rather than other two postural tasks. This finding was consistent with those reported by Etemadi [18] et al. and Salavati [19] et al. Both of these two studies showed the LBP group had worse performance in cognitive task compared to the control group. In Etemadi [18] et al’s study the reaction times of cognitive task of LBP participants were slower than those of the controls in all conditions. While in Salavati [19] et al’s study more cognitive errors were found in the LBP group than control group when the cognitive task was most difficult with higher postural difficulty. The one-leg stance has been found to be a more challenging balance condition [22] because it may require more cognitive resources to maintain balance compared with that of other postural tasks (eyes-open and eyes-closed). The one-leg stance would become more difficult when carrying out two tasks simultaneously due the limits of cognitive capacity [43]. In the present study, older participants with LBP showed decreased postural stability compared with that in control group at three levels of postural tasks, and cognitive performance became worse only in the one-leg stance. The potential reason was that increasing the difficulty of the postural/cognitive task in the dual-task would result in insufficient cognitive resources to be allocated to posture tasks for people with LBP, especially if the postural task was more difficult [44].

In the present study, significant associations in the single task were shown only in the eye-closed condition. These findings are supported by a study suggesting vision to be an important risk factor for falling [45]. More associations between sway area/sway length and the number of falls were significant in dual-task conditions than in single-task conditions. This finding is consistent with that in a study reporting dual-task testing to be more strongly associated with fall risk than single-task testing [46]. The reason for these findings is that the dual-task paradigm is similar to the activities of daily living, which require cognitive and motor tasks to be undertaken simultaneously.

Our study addresses several of the gaps in knowledge and limitations of previous research in this area. Few studies have employed a dual-task model to assess PC in older women with LBP. In particular, we combined different levels of posture tasks and cognitive tasks. Given the importance of a dual-task performance for independent living in old age, this emerging research area, which relates attention and PC, has become a “hotspot”. However, PC impairment and body instability in older adults with LBP resulting from deficits in the allocation of attention have been considered only recently. Our results suggested that LBP seems to have an interaction with cognitive functions, and sequentially result in postural instability.

Our study had five main limitations. First, our study had a cross-sectional design. Prospective cohort studies are needed to investigate the causal relationship between cognitive loads in postural tasks and the number of falls. Second, we used only behavioral PC to examine the modulation effect of cognitive loads on PC. Future studies should employ electromyography and/or electroencephalography to explore the underlying neural mechanism. Third, this study do not assess the effects of cognitive load on dynamic balance, which also reflect the postural control of older people. Fourth, always starting at 100 in the serial-7 s arithmetic task would lead to a learning effect, which make the task easier as participants progressed through testing. The start number should be selected at random in the range of 80 to 99 to avoid the learning effect in the future study. Fifth, both cognitive loads and articulation in the dual tasks could contribute to the changes of postural control [47]. In the present study participants verbalized their answers in both two cognitive tasks, so the difference between two cognitive tasks could tease out the effect of articulation. However, the single task was conducted without any cognitive task. The difference of postural performance between the dual task and single task could be attributed to a combination effect of cognitive loads and the motor aspect of articulation. In the future study, a postural task combined with an articulation task should be used as the control task. Finally, all the participants recruited in the present study were female, which hampered the generalizability of our findings.

## Conclusions

We revealed that, compared with control group, older women with LBP showed poor PC regardless of the difficulties of postural tasks (especially during concurrent postural and cognitive tasks). Our findings shed light on how cognitive loads modulate PC and suggest that dual-task training could be an effective rehabilitation intervention for older women with LBP.

## Data Availability

The datasets used and/or analysed during the current study are available from the corresponding author upon reasonable request.

## Abbreviations

LBP: Low back pain
PC: Postural control
MMSE: Mini-Mental State Examination
MoCA: Montreal Cognitive Assessment
VAS: Visual analog scale
AP: Anteroposterior
ML: Mediolateral
SO: Single task with eyes-open
SC: Single task with eyes-closed
Sleg: Single task with one-leg stance
DO1: Dual task (postural task and auditory arithmetic task) with eyes-open
DC1: Dual task (postural task and auditory arithmetic task) with eyes-closed
Dleg1: Dual task (postural task and auditory arithmetic task) with one-leg stance
DO2: Dual task (postural task and Serial-7 s arithmetic task) with eyes-open
DC2: Dual task (postural task and Serial-7 s arithmetic task) with eyes-closed
Dleg2: Dual task (postural task and Serial-7 s arithmetic task) with one-leg stance
